# Integrated Metabolomic and Transcriptomic Analyses Identify Elevated Tryptophan Metabolism and Altered Sugar Homeostasis Associated with Honeybee Jujube Flower Disease

**DOI:** 10.3390/biology15151236

**Published:** 2026-07-26

**Authors:** Miaoran Zhang, Yali Du, Yumeng Zhang, Kai Xu, Yusuo Jiang, Qingsheng Niu

**Affiliations:** 1 Jilin Provincial Key Laboratory of Bee Genetics and Breeding, Apiculture Science Institute of Jilin Province, Jilin 132108, China; mrzhang20@mails.jlu.edu.cn (M.Z.); xukaiyuzhong@126.com (K.X.); 13943233663@163.com (Q.N.); 2 College of Animal Science and Technology, Jilin Agricultural University, Changchun 130118, China; 18637486236@163.com; 3 College of Animal Science, Shanxi Agricultural University, Jinzhong 030801, China; ysjiang@sxau.edu.cn

**Keywords:** *Apis mellifera*, differential metabolites, gene-set enrichment analysis, metabolic remodeling, multi-omics integration, widely targeted metabolomics

## Abstract

Jujube flower disease (JFD) is a recurrent disorder affecting honeybees during the flowering period of *Ziziphus jujuba*, but its molecular basis remains unclear. To characterize JFD-associated molecular changes, we integrated widely targeted metabolomics and RNA sequencing (RNA-seq) in exposed asymptomatic jujube flower foragers (HC) and in bees showing characteristic JFD symptoms. Filtered metabolomic profiles showed clear separation between HC and JFD samples and revealed two major metabolite-level alterations. Tryptophan metabolism was the strongest enriched pathway among differentially abundant metabolites (Rich Factor = 14/16; FDR = $9.15\times 10^{-7}$), whereas metabolites decreased in JFD were enriched mainly in galactose metabolism (Rich Factor = 7/16; FDR = $1.23\times 10^{-4}$), amino sugar and nucleotide sugar metabolism (Rich Factor = 5/15; FDR = 0.00762), biosynthesis of nucleotide sugars (Rich Factor = 4/15; FDR = 0.0318), and starch and sucrose metabolism (Rich Factor = 3/8; FDR = 0.0318). RNA-seq detected 10,259 genes and identified 23 differentially expressed genes under the selected threshold, including 20 increased and 3 decreased genes in JFD bees. Although individual-gene differential expression was limited, preranked gene-set enrichment analysis identified moderate negative enrichment of the sugar supply/storage gene set (NES = −1.96; FDR = 0.024). The tryptophan/aromatic amino acid gene set was not enriched at the transcriptomic level, indicating that the tryptophan-related alteration was mainly observed at the metabolite level. Within these gene sets, HK showed the largest downward RNA-seq trend among the five displayed sugar-axis enzyme genes and a directionally concordant qRT-PCR trend; on this basis, HK was retained only as a candidate for future functional validation. Overall, these findings suggest that JFD is associated with altered tryptophan-related metabolism and reduced carbohydrate/nucleotide sugar-related metabolite abundance, with partial transcriptomic support for altered sugar supply and storage. This molecular profile provides candidate pathways and genes for future mechanistic investigation rather than established biomarkers, regulatory mechanisms, or evidence of disease causation.

## Simple Summary

Jujube flower disease reduces bee health during the flowering period of jujube trees, but its molecular basis remains unclear. This study identified two major disease-associated molecular alterations: increased tryptophan-related metabolites and reduced carbohydrate- and nucleotide sugar-related metabolites. Transcriptomic gene-set analysis provided moderate support for altered sugar supply and storage, and HK was therefore retained only as a follow-up candidate, based on it having the largest downward RNA-seq trend among the displayed sugar-axis enzyme genes and its consistent qRT-PCR trend.

## 1. Introduction

The flowering stage of jujube (*Ziziphus jujuba*) is a critical period for pollination and bee–flower interactions, and studies across *Ziziphus* species indicate close links among insect pollination, nectar secretion, and apicultural value [1,2,3]. As a nectar-producing crop, jujube attracts large numbers of honeybee foragers during bloom, making this period an important exposure window for bee colonies placed in jujube-producing areas. However, in the major jujube-producing regions of northern China, honeybees often exhibit abnormal behavior, increased mortality, and colony decline after collecting jujube flower nectar, a bloom-associated condition commonly referred to as jujube flower disease (JFD). This problem has long constrained colony use efficiency and bee-product yield during the flowering period. Previous work has shown significant differences in gut microbial composition and diversity between JFD bees and healthy individuals, suggesting that JFD is not merely a field phenomenon but is accompanied by detectable biological alterations [4]. A subsequent midgut-focused study further reported midgut structural damage, reduced antioxidant and detoxification capacity, and transcript-level responses in JFD bees [5]. These findings indicate that JFD is accompanied by measurable gut-associated structural, biochemical, and transcriptomic changes. However, previous studies have primarily examined intestinal-localized alterations and therefore cannot determine whether these changes are accompanied by systemic metabolic remodeling across the whole organism or whether gut responses represent the major molecular features associated with JFD. Therefore, the molecular alterations beyond the gut and their relationship to broader metabolic changes remain insufficiently defined. The present study extends previous work by integrating whole bee metabolomics with matched transcriptomic analysis to characterize JFD-associated metabolic alterations and their transcript-level support.

The molecular basis of JFD remains insufficiently defined. Earlier explanations focused mainly on relatively high levels of alkaloids, potassium ions, or sugars in jujube nectar or the surrounding environment, but these hypotheses were based largely on empirical observation or single-factor inference and have not identified the key molecular abnormalities involved in disease development [4]. These proposed factors are chemically and physiologically distinct and would be expected to produce different downstream biochemical signatures related to xenobiotic exposure, ionic or osmotic stress, nutrient availability, or energy metabolism. Metabolomic profiling is therefore suitable for this question because it can capture a broad range of small-molecule and pathway-level changes in the same biological samples. Although metabolomics alone cannot establish disease causation or directly test all proposed factors, it provides a systematic approach for identifying the metabolic alterations most prominently associated with JFD. Previous JFD studies have placed the disease within a biological context involving intestinal homeostasis, detoxification, energy metabolism, and abnormal locomotor or flight-related behavior. Because JFD occurs during the jujube flowering period and can lead to forager loss, colony decline, and reduced colony-use efficiency, it is relevant to both insect physiology and apicultural practice. However, how the previously described intestinal and transcriptomic responses are connected to broader metabolic changes and host gene expression programs remains insufficiently defined.

Although previous studies have indicated that the activity of chitinase and carboxylesterase (CarE) is associated with the molecular basis of JFD [5], carbohydrate metabolism during jujube nectar foraging may also be relevant to this disease. Honeybees rely heavily on nectar and pollen for nutrition and energy, with nectar serving as the major immediate carbohydrate source [6,7]. In foraging honeybees, hemolymph sugar levels are associated with metabolic rate and trehalose synthesis capacity [8], and parasitic infection can reduce hemolymph sugar levels, indicating that carbohydrate status is linked to energetic and infection-related stress physiology [9]. In addition, honeybee flight relies primarily on hexose oxidation [10], and flight-associated metabolic capacity is closely related to glycolytic enzymes such as hexokinase, phosphofructokinase, and pyruvate kinase [11]. At the colony level, nectar foraging behaves as a supply-driven system, underscoring the importance of resource availability for colony maintenance and food storage [12]. At the individual level, foraging behavior is jointly regulated by the quality and quantity of nectar reward, indicating that sugar reward is a key determinant of foraging decisions [13,14]. Honeybee nutritional state is also closely linked to circulating carbohydrates, trehalose homeostasis, and feeding-related physiology [6,7]. Genomic and comparative physiological studies further indicate that honeybees have evolved a specialized metabolic system adapted to a high-sugar diet [15]. Given the nectar-foraging context of JFD and its typical symptoms of abnormal crawling and impaired flight, carbohydrate acquisition, storage, and utilization provide a relevant metabolic entry point for investigating JFD-associated physiological changes. This focus does not exclude the possible involvement of lipid metabolism, oxidative phosphorylation, or other energy-related processes. However, whether carbohydrate metabolic alteration contributes to JFD development or reflects a secondary physiological response remains unclear.

At the same time, JFD-associated metabolic changes may extend beyond carbohydrate metabolism. Previous studies have linked the honeybee gut microbiota to host nutrient processing, metabolic state, behavior, and neurophysiology [16,17,18,19]. Nutrient-state pathways, including IIS, TOR, and FoxO-related signaling, are also closely associated with insect energy homeostasis, behavioral regulation, and foraging-state transitions in honeybees [20,21,22,23]. These observations provide a broader biological context in which JFD-associated metabolic disturbance may involve amino acid metabolism, host–microbiota interactions, and nutrient-state regulation. However, which metabolite pathways are most prominently altered in JFD and whether these changes are accompanied by coordinated host transcriptomic responses remain unclear.

Analysis at a single omics layer is often insufficient to identify disease-relevant molecular programs. Metabolomics captures downstream biochemical changes that may not necessarily be mirrored by differential gene expression, whereas RNA-seq measures transcript-level remodeling of functional gene programs [24]. Multi-omics integration based on matched samples may therefore help distinguish metabolite-level alterations from changes that also show transcript-level support and may provide biologically grounded candidates for follow-up validation [25,26].

Although previous studies have identified alterations in gut microbiota composition, intestinal morphology, antioxidant capacity, and intestinal gene expression in JFD bees, these findings mainly describe gut-associated responses and do not establish whether JFD involves broader metabolic remodeling at the whole-organism level. Moreover, whether metabolite-level alterations are accompanied by corresponding transcriptomic changes remains unclear. Therefore, an integrated analysis of metabolic and transcriptional alterations is needed to better characterize the molecular features associated with JFD. Based on these observations, we hypothesized that JFD is associated with whole-bee metabolic remodeling and that a subset of metabolite alterations would be accompanied by corresponding transcriptomic changes.

In this study, we integrated widely targeted metabolomics and RNA-seq to compare HC and JFD bees. Our analysis identified metabolic alterations associated with JFD, evaluated whether these alterations were accompanied by transcript-level changes in functionally related gene sets, and explored candidate genes encoding enzymes directly linked to measured sugar-related metabolites based on expression trends, direct biochemical relationships, and qRT-PCR validation. These findings provide a more balanced molecular view of JFD-associated metabolic remodeling.

## 2. Materials and Methods

### 2.1. Sample Collection

At the early flowering stage (approximately 23 May), 30 healthy *Apis mellifera ligustica* colonies unexposed to jujube blossoms were transferred to a jujube orchard near Yongyou Village, Peigou Township, Shilou County, Shanxi Province (37°10′6.24″ N, 110°44′42.6″ E). During peak jujube bloom (10–12 June), JFD was classified using a field-defined syndromic presentation consistent with previous reports [4,5]. Bees exhibiting an arched and constricted abdomen, uncoordinated crawling, and impaired flight were randomly collected at hive entrances and assigned to the JFD group, whereas active nectar-foraging bees collected from jujube blossoms without these signs were assigned to the HC group. In the present study, the HC designation was used operationally to refer to exposed asymptomatic foragers collected under the same bloom conditions rather than to unexposed healthy bees. No pathogen-specific diagnostic testing or pesticide/toxicant residue analysis was performed. All collected samples were immediately snap-frozen in liquid nitrogen and stored at −80 °C for widely targeted metabolomics, RNA-seq, and qRT-PCR analyses. The metabolomic analysis included eight HC and eight JFD individuals, with one individual sampled from each of 16 different colonies. Before the metabolomic results were examined, three HC and three JFD individuals were selected for RNA-seq analysis by simple random sampling within each group. This procedure maintained equal group representation, and the six selected individuals originated from six different colonies. Thus, no colony contributed more than one individual to either omics dataset, and each individual bee was treated as one biological replicate in the corresponding analysis.

### 2.2. Metabolite Extraction and Widely Targeted Metabolomics Analysis

Widely targeted metabolomics was used to detect metabolites in whole bee samples. Samples were removed from −80 °C storage, thawed on ice, and processed under low-temperature conditions. Each sample was thoroughly ground in liquid nitrogen, and 20 ± 1 mg of tissue was weighed into a centrifuge tube. Then, 400 µL of 70% aqueous methanol extraction solution containing internal standards was added. After vortexing at 1500 rpm for 5 min, the samples were incubated in an ice bath for 15 min. Samples were centrifuged at 4 °C and 12,000 rpm for 10 min, and 300 µL of supernatant was transferred to a new centrifuge tube. After storage at −20 °C for 30 min, the samples were centrifuged again at 4 °C and 12,000 rpm for 3 min, and 200 µL of supernatant was collected for UPLC-MS/MS analysis.

Metabolite detection was performed on a UPLC-MS/MS platform. Equal volumes of each sample extract were pooled to generate a quality control (QC) sample, which was first subjected to widely targeted liquid chromatography–quadrupole time-of-flight tandem mass spectrometry (LC-QTOF-MS/MS) analysis. Biological samples were analyzed in a randomized injection order, with HC and JFD samples distributed throughout the analytical sequence. Quality control was evaluated using pooled QC samples inserted at regular intervals during the LC-MS/MS run to monitor analytical stability and potential instrumental drift. The widely targeted metabolomic analysis was performed using an ExionLC AD UPLC system coupled to a TripleTOF 6600 mass spectrometer (AB SCIEX, Framingham, MA, USA), with an ACQUITY HSS T3 column (2.1×100 mm, 1.8 µm; Waters Corporation, Milford, MA, USA). Mobile phase A was 0.1% formic acid in water, and mobile phase B was 0.1% formic acid in acetonitrile. The column temperature was 40 °C, the flow rate was 0.4 mL/min, and the injection volume was 5 µL. The gradient elution program was as follows: 0 min, A/B = 95/5; 11.0 min, A/B = 10/90; 12.0 min, A/B = 10/90; 12.1 min, A/B = 95/5; and 14.0 min, A/B = 95/5. Mass spectrometry data were acquired in both positive and negative ion modes. The ion source temperature was 500 °C, and the ion spray voltage was 5500 V in positive mode and −4500 V in negative mode.

The widely targeted metabolomic data were qualitatively annotated using the in-house MWDB database, integrated public databases (Metlin, the Human Metabolome Database (HMDB), and the Kyoto Encyclopedia of Genes and Genomes (KEGG)), prediction-based annotation resources, and MetDNA. Based on the detected multiple-reaction ion pairs, retention times, and annotation information, a project-specific targeted library was then established, and all samples were quantified on a UPLC-QTRAP-MS/MS platform using multiple reaction monitoring (MRM) mode. The quantitative platform consisted of an ExionLC AD UPLC system coupled to a QTRAP mass spectrometer 6500. Chromatographic separation was performed on a Waters (Milford, MA, USA) ACQUITY UPLC HSS T3 C18 column (1.8 µm, 2.1×100 mm), using the same mobile phases and gradient as in the widely targeted metabolomic analysis, with a flow rate of 0.4 mL/min, a column temperature of 40 °C, and an injection volume of 2 µL. Raw data were processed using Analyst v1.6.3, and chromatographic peak areas were integrated and corrected with MultiQuant (SCIEX, Framingham, MA, USA) to obtain relative metabolite abundances. Quality control was evaluated using pooled QC samples injected throughout the LC-MS/MS run. For the 2382 quantified metabolite features, the median QC relative standard deviation (RSD) was 7.77%, and 95.4% of features had QC RSD ≤ 30%. Pairwise correlations among QC samples were high (r = 0.9974–0.9982). Eight internal standards were monitored, with CV values ranging from 0.90% to 2.33% (median CV = 1.52%), supporting analytical stability. The names and CV values of the eight internal standards are provided in Supplementary Table S1. All biological samples were analyzed in a single batch; therefore, no between-batch correction was required. Downstream analyses were performed on log_2_-transformed, quality-controlled abundance values. Metabolite annotations were assigned based on MS/MS spectral and database matching, with annotation sources including MS2-mHK6.0, MS2-MetDNA, MS2-MedDB2.0, and in silico MS2 matching. Unless otherwise indicated, metabolites were treated as putatively annotated compounds rather than authentic-standard-confirmed identifications.

### 2.3. Metabolite Annotation, Filtering, and Differential Analysis

The raw metabolite quantification matrix included compound names, classification information, molecular formulas, sample peak areas, and annotations from KEGG, HMDB, PubChem, ChEBI, and Metlin. To further clarify the filtering procedure, a two-stage annotation-based filtering workflow was applied. First, features were retained when their annotations showed compatibility with host-associated metabolic processes based on database evidence, structural information, or metabolite-class characteristics. Features lacking host-associated evidence or carrying clear exogenous annotations, KEGG drug identifiers, or irrelevant chemical categories were excluded. This step retained 1183 of the 2382 quantified features. Second, 38 retained annotations were manually removed because they were considered likely plant-derived or exposure-related compounds, including flavonoid/lignan-related, drug-like, antibiotic, steroid, and contaminant annotations. The final set of 1145 filtered annotated metabolites was used for downstream PCA, differential abundance analysis, visualization, and KEGG enrichment analysis. Because the biological origin of each metabolite cannot be definitively assigned from the present MS/MS- and database-based annotation workflow alone, the retained features are referred to as filtered annotated metabolites.

The relative abundance matrix used peak areas as input. To reduce scale differences and improve statistical stability, peak areas for all samples were first ${log}_{2}$-transformed. Raw peak areas below 1 were truncated to 1 before transformation to avoid invalid logarithms caused by zeros or extremely low values.

Principal component analysis was performed using the retained metabolite matrix. Before analysis, the sample-by-metabolite matrix was standardized by metabolite, and the first two principal components were extracted for visualization. Differential metabolite analysis was performed on the ${log}_{2}$-transformed metabolite matrix. For each metabolite, a linear model was fitted as abundance∼group, where abundance represents the ${log}_{2}$ relative abundance of the metabolite across samples and group was coded with HC as the reference group and JFD as the comparison group. Accordingly, the group coefficient represents the ${log}_{2}$ fold change in JFD relative to HC. *p* values for all metabolites were adjusted for multiple testing using the Benjamini–Hochberg method to obtain FDR values. Significant differential metabolites were defined as those with FDR < 0.05, which was used as the primary statistical threshold to control the expected false-discovery rate across metabolite-wise tests. In volcano plots and focused screening of differential metabolites, an additional effect-size threshold of |log_2_FC| ≥ 0.5 was applied to highlight metabolites with both statistical support and a minimum magnitude of change. This effect-size threshold did not replace FDR-based significance testing. Heatmaps of differential metabolites were generated from the matrix of significant differential metabolites. Metabolites were ranked first by ascending FDR and then by descending |log_2_FC|, and the top 40 metabolites were selected only for visualization. Each metabolite was row-wise z-score standardized across samples to display relative abundance patterns.

### 2.4. KEGG Pathway Enrichment Analysis

KEGG enrichment analysis was restricted to the filtered annotated metabolites retained after annotation-based filtering. The background set was defined as all filtered annotated metabolites included in the differential abundance analysis. Foreground sets were defined separately as all significant differentially abundant filtered metabolites, significantly increased filtered metabolites, and significantly decreased filtered metabolites. Pathway enrichment was evaluated using a right-tailed hypergeometric test to determine whether metabolites assigned to each KEGG pathway were overrepresented in the corresponding foreground set relative to the background set. Enrichment *p* values for all pathways were adjusted using the Benjamini–Hochberg method to obtain FDR values.

### 2.5. RNA Extraction

Total RNA was extracted from six matched metabolomic samples (HC1, HC2, HC3, JFD1, JFD2, and JFD3) using TRIzol^TM^ reagent (Invitrogen, Carlsbad, CA, USA) from whole bee samples. RNA quality and integrity were detected on a Qubit^®^2.0 Flurometer (Invitrogen, Carlsbad, CA, USA) and an Agilent Bioanalyzer 2100 system (Agilent Technologies, Santa Clara, CA, USA). cDNA libraries were generated using NEBNext^®^ Ultra^TM^ RNA Library Prep Kit for Illumina^®^ (New England Biolabs, Ipswich, MA, USA) and sequenced on the Illumina (San Diego, CA, USA) platform by Metware Biotechnology Co., Ltd (Wuhan, China).

### 2.6. RNA-Seq Processing and Differential Expression Analysis

The raw RNA-seq data consisted of 150 bp paired-end reads generated on the Illumina platform. Raw reads were first processed using fastp for quality control, including adapter removal and filtering of reads with excessive proportions of N bases or low-quality bases. Filtered clean reads were aligned to the western honeybee reference genome Amel_HAv3.1. After alignment, total reads, mapped reads, uniquely mapped reads, multiple mapped reads, and the distribution across exon, intron, and intergenic regions were summarized to evaluate data utilization and annotation consistency. On average, each sample generated 49.91 million clean reads, corresponding to 7.49 Gb of clean sequence data. The mean Q30 value was 93.90%, the mean overall mapping rate was 93.05%, and the mean uniquely mapped rate was 91.93%. Sample-level sequencing and alignment quality metrics are provided in Supplementary Table S2.

Gene expression quantification was performed using featureCounts to generate a gene-level raw count matrix. The raw count matrix was used for differential expression analysis, whereas the voom-transformed expression matrix was used for PCA, targeted heatmaps, and subsequent sugar-related gene–metabolite analysis. The RNA-seq samples included in downstream analysis were the six samples shared with the metabolomics dataset: HC1, HC2, HC3, JFD1, JFD2, and JFD3. To reduce the influence of lowly expressed genes, genes with CPM ≥ 1 in at least two samples were retained, resulting in 10,259 genes for downstream limma-voom analysis.

Differential expression analysis was conducted using the raw count matrix. RNA-seq sample columns were first extracted to construct a count matrix with genes as rows and samples as columns, and a design matrix was generated using model.matrix(group), with HC set as the reference group. To reduce the impact of lowly expressed genes, counts per million (CPM) were calculated based on library size, and genes with CPM ≥1 in at least two samples were retained. The retained raw counts were then processed using limma::voom() for mean-variance modeling, yielding ${log}_{2}$-counts-per-million expression values and observation-level precision weights. Linear model fitting was performed using limma::lmFit(), followed by empirical Bayes variance shrinkage with limma::eBayes(). Multiple-testing correction was performed using the Benjamini–Hochberg method.

### 2.7. Validation of Differentially Expressed Genes by qRT-PCR

Total RNA for qRT-PCR validation was extracted from whole bee samples from the same sampling batch and sample type used for RNA-seq analysis, using TRIzol^TM^ reagent (Invitrogen, Carlsbad, CA, USA), and was then assessed for RNA purity and concentration.

First-strand complementary DNA (cDNA) was synthesized from 1 µg of total RNA using the PrimeScript^TM^ RT Reagent Kit with gDNA Eraser (Perfect Real Time, TaKaRa, Dalian, China) and then diluted five times with nuclease-free water. Then, qRT-PCR analysis was performed on a 7500 Real-time PCR system (ABI, Foster City, CA, USA) using TB Green^®^ Premix Ex Taq^TM^ (Tli RNaseH Plus, TaKaRa, Biotechnology Co., Ltd., Dalian, China) in a 15 µL reaction containing 1.5 µL of cDNA, 0.6 µL of each primer (10 µM), 7.5 µL of SYBR Premix Ex Taq II (2×), 0.3 µL of ROX Reference Dye II (2×), and 4.5 µL of ddH_2_O. The procedure included a reaction at 95 °C for 30 s, followed by 45 cycles at 95 °C for 5 s, and another reaction at 62 °C for 34 s. At the end of the analysis, melt curves were generated using the following conditions: 95 °C for 15 s, 62 °C for 1 min, and 95 °C for 15 s. Three technical replicates were performed for each biological sample. Specific primers for qRT-PCR were designed using the Primer 3.0 plus server (https://www.primer3plus.com/index.html (accessed on 6 May 2022)), and the primer sequences used in this study are shown in Table 1. All data obtained by qRT-PCR were analyzed using 7500 Software (v2.0), and the relative expression was normalized to Arp1 (LOC406122) mRNA using the comparative $2^{-\Delta \Delta Ct}$ method. Each individual bee was treated as an independent biological sample.

### 2.8. Functional Gene-Set Analysis and Targeted Transcriptome Visualization

To analyze the transcriptome around the major directions of change suggested by the metabolomic results, four functionally related gene sets were constructed in this study, representing tryptophan/aromatic amino acids, sugar supply/storage, carbon flow, and juvenile hormone (JH)/IIS/TOR/FoxO-related nutrient signaling. To assess overall expression shifts in these gene sets at the transcriptomic level, preranked gene-set enrichment analysis (preranked GSEA) was performed on the differential expression results. The ranking statistic was defined as the product of $sign({log}_{2}FC)$ and the negative base-10 logarithm of the FDR after adding $1\times 10^{-300}$, integrating both direction of change and statistical strength. All genes were ranked according to this statistic and analyzed using fgsea, and the normalized enrichment score (NES), FDR, and leading-edge genes were recorded.

For transcriptomic visualization, this study did not use genome-wide clustered heatmaps. Instead, targeted heatmaps were generated around functionally related gene sets directly connected to the metabolomic findings. Heatmaps mainly displayed genes from the sugar supply/storage-, tryptophan/aromatic amino acid-, and carbon flow-related sets. For each heatmap, genes were first restricted to members of the corresponding gene set, then ranked by $\left|{log}_{2}FC\right|$, with priority given to genes with smaller FDR values. For the carbon-flow-related set, priority was also given to genes whose annotations matched pyruvate-, propanoate-, or branched-chain amino acid-related descriptions. Heatmap expression matrices were derived from voom-transformed expression values and were row-wise z-score standardized before plotting.

### 2.9. Exploratory Direct Enzyme–Metabolite Pair Analysis

To further examine gene–metabolite covariation within the sugar supply/storage program, we focused on direct enzyme–metabolite relationships between genes in the sugar supply/storage gene set and measured sugar-related metabolites. This analysis was not designed as a global correlation screen. Instead, representative pairs were selected according to direct biochemical correspondence, defined as cases in which the encoded enzyme was directly linked to the measured metabolite as a substrate or product within the sugar supply/storage-related pathway context.

RNA-seq expression data were extracted from the raw quantification matrix and analyzed in the six samples directly matched to the metabolomics dataset: HC1, HC2, HC3, JFD1, JFD2, and JFD3. Counts per million (CPM) values were first calculated from the count matrix, genes with CPM ≥ 1 in at least two samples were retained, and the matrix was then transformed to log2-counts-per-million expression values using limma::voom(). The metabolomic analysis used the corresponding log2-transformed, quality-controlled relative-abundance matrix from the single analytical batch; no batch-effect correction was applied.

Five direct enzyme–metabolite pairs were selected for visualization: Pgm1–D-glucose-6-phosphate, trehalase–D-glucose, Pgi–D-glucose-6-phosphate, Tps1–UDP-glucose, and HK–D-glucose. These pairs were selected because the encoded enzymes are directly connected to the displayed metabolites within sugar interconversion, trehalose metabolism, nucleotide sugar metabolism, or glucose phosphorylation. Spearman rank correlation was calculated across the six matched samples for each displayed pair and was used only as an exploratory descriptor of sample-level covariation. Nominal *p* values were shown as descriptive annotations for the plotted pairs but were not used to define statistical significance, to screen candidate genes, or to infer causal regulation. All individual samples were labeled in the scatter plots to allow for visual inspection of sample-level influence.

For the same five displayed pairs, leave-one-out sensitivity analysis was performed by removing one sample at a time and recalculating Spearman’s ρ using the remaining five samples. This analysis was used only to evaluate whether the direction of the displayed correlation was sensitive to individual samples and was not treated as an independent statistical test.

Expression distributions of the five displayed genes were visualized using voom-derived log2 counts per million (logCPM) values as boxplots overlaid with individual sample points. The corresponding RNA-seq log_2_FC and adjusted *p* values from the limma-voom differential-expression analysis were displayed in each panel.

### 2.10. Software

Metabolomics reanalysis was performed in Python 3.13.5, mainly using pandas 2.3.3, NumPy 2.2.6, SciPy 1.16.1, statsmodels 0.14.5, scikit-learn 1.7.1, matplotlib 3.10.7, and seaborn 0.13.2. Downstream transcriptomic analyses were performed in R 4.5.3, mainly using readxl 1.4.5, limma 3.66.0, fgsea 1.36.2, ggplot2 4.0.2, dplyr, tidyr, stringr, ggrepel 0.9.7, openxlsx 4.2.8.1, and patchwork 1.3.2. Principal component analysis was implemented using either scikit-learn or stats::prcomp(). Volcano plots, heatmaps, enrichment plots, gene–metabolite correlation heatmaps and candidate-gene expression plots were generated using ggplot2, and composite figures were assembled with patchwork. Expression plots for displayed sugar-axis genes and putative follow-up genes were based on voom-derived logCPM values and displayed as boxplots overlaid with scatter points.

### 2.11. Statistical Analysis

For metabolomic data, differential metabolites were identified using a linear model followed by Benjamini–Hochberg false discovery rate (FDR) adjustment; metabolites with FDR < 0.05 were considered statistically significant, and an absolute log_2_ fold change ≥ 0.5 was additionally used for visualization and focused screening. KEGG pathway enrichment was evaluated by a hypergeometric test with Benjamini–Hochberg correction, and pathways with FDR < 0.05 were regarded as significantly enriched. For RNA-seq data, differential expression analysis was performed using the limma-voom framework with empirical Bayes moderation, and multiple testing was controlled by the Benjamini–Hochberg method (FDR < 0.05). Preranked gene-set enrichment analysis (GSEA) was conducted using the fgsea algorithm, and gene sets with FDR < 0.05 were considered significantly enriched. For exploratory direct enzyme–metabolite pair analysis, Spearman rank correlation was calculated for each selected pair across matched samples; the resulting correlation *p* values were used only as descriptive annotations, not as formal significance thresholds, and leave-one-out sensitivity analysis was performed to assess robustness. Principal component analysis (PCA) and all other visualizations (volcano plots, heatmaps, boxplots, scatter plots) were descriptive and involved no additional hypothesis testing.

## 3. Results

### 3.1. Widely Targeted Metabolomics Reveals Broad Metabolic Differences Between HC and JFD Bees

**Figure 1:**
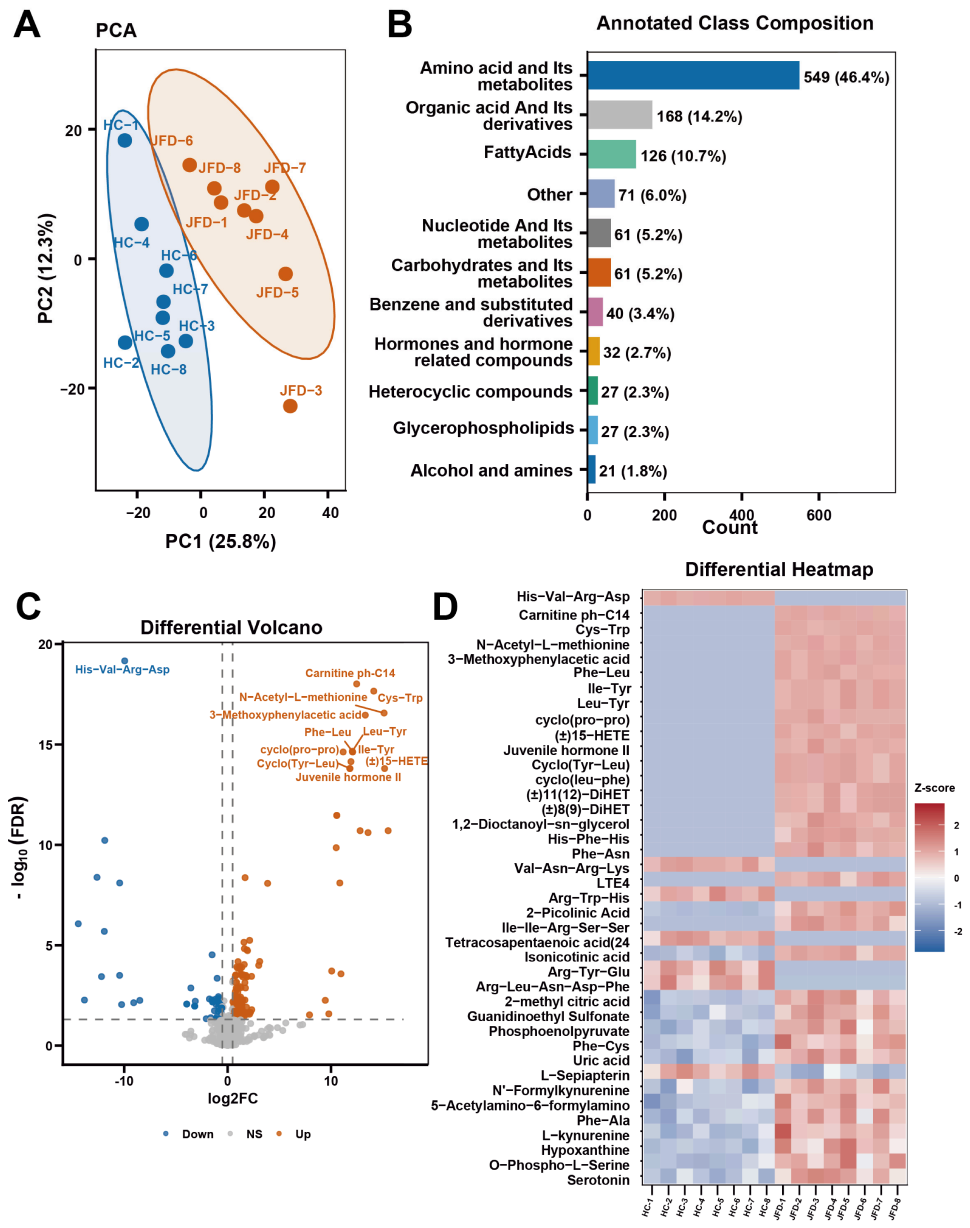
Widely targeted metabolomics reveals broad metabolite abundance differences between HC and JFD bees. (**A**) Principal component analysis (PCA) based on the 1145 filtered annotated metabolites retained after annotation-based filtering. Ellipses indicate the 95% confidence regions for each group. Blue points and ellipses represent HC samples, whereas orange points and ellipses represent JFD samples. (**B**) Annotated classes and composition of the filtered metabolite set. Numbers indicate metabolite counts, and percentages are shown in parentheses. Bar colors distinguish the annotated metabolite classes and do not represent metabolite abundance, differential direction, or statistical significance. (**C**) Volcano plot of differentially abundant filtered metabolites between HC and JFD. Metabolites with FDR < 0.05 are highlighted, and those meeting both FDR < 0.05 and |log_2_FC| ≥ 0.5 were considered the major differential metabolites for focused visualization. Representative metabolites are labeled. The horizontal dashed line indicates FDR = 0.05, the two flanking vertical dashed lines indicate log_2_FC = −0.5 and 0.5, and the central vertical dashed line indicates log_2_FC = 0. Orange, increased in JFD; blue, decreased in JFD; gray, not significant. (**D**) Heatmap of the top 40 significant differentially abundant filtered metabolites ranked by FDR and |log_2_FC|. Metabolite abundances were row-wise z-score standardized before visualization. Red indicates relatively higher abundance, and blue indicates relatively lower abundance across samples.

To characterize the global metabolite abundance changes associated with jujube flower disease, we performed widely targeted metabolomic profiling in exposed asymptomatic foragers (HC) and bees with jujube flower disease (JFD). In total, 2382 metabolites were quantified, of which 1145 filtered annotated metabolites were retained after annotation-based filtering for downstream analysis (Supplementary Table S3). Principal component analysis based on these filtered annotated metabolites showed separation between HC and JFD samples, and the 95% confidence ellipses further supported distinct group distributions (Figure 1A). An additional PCA including the pooled QC injections showed close clustering of the three QC injections, consistent with stable analytical performance during LC-MS/MS acquisition (Supplementary Figure S1).

The filtered annotated metabolite set was dominated by amino acids and their metabolites (549, 46.4%), followed by organic acids and their derivatives (168, 14.2%) and fatty acids (126, 10.7%), while nucleotide-related, carbohydrate-related, hormone-related, and other annotated classes were also represented (Figure 1B, Supplementary Figure S2). These data indicate that the filtered metabolite matrix covered a broad range of annotated biochemical classes.

We next examined metabolite abundance differences between the two groups. Among the 1145 filtered annotated metabolites, 237 met the significance threshold of FDR < 0.05, and 220 of these also satisfied the effect-size criterion of |log_2_FC| ≥ 0.5. Relative to HC, 180 metabolites were increased and 57 were decreased in JFD bees based on FDR < 0.05. The complete list of significantly altered metabolites is provided in Supplementary Table S4. The volcano plot and the heatmap of the top 40 significant metabolites ranked by FDR and |log_2_FC| further illustrate the broad metabolic divergence between the two groups (Figure 1C,D). Together, these data indicate that JFD is associated with widespread remodeling of the retained metabolite profile.

### 3.2. KEGG Analysis Shows Elevated Tryptophan and Reduced Sugar Pathways in JFD Bees

**Figure 2:**
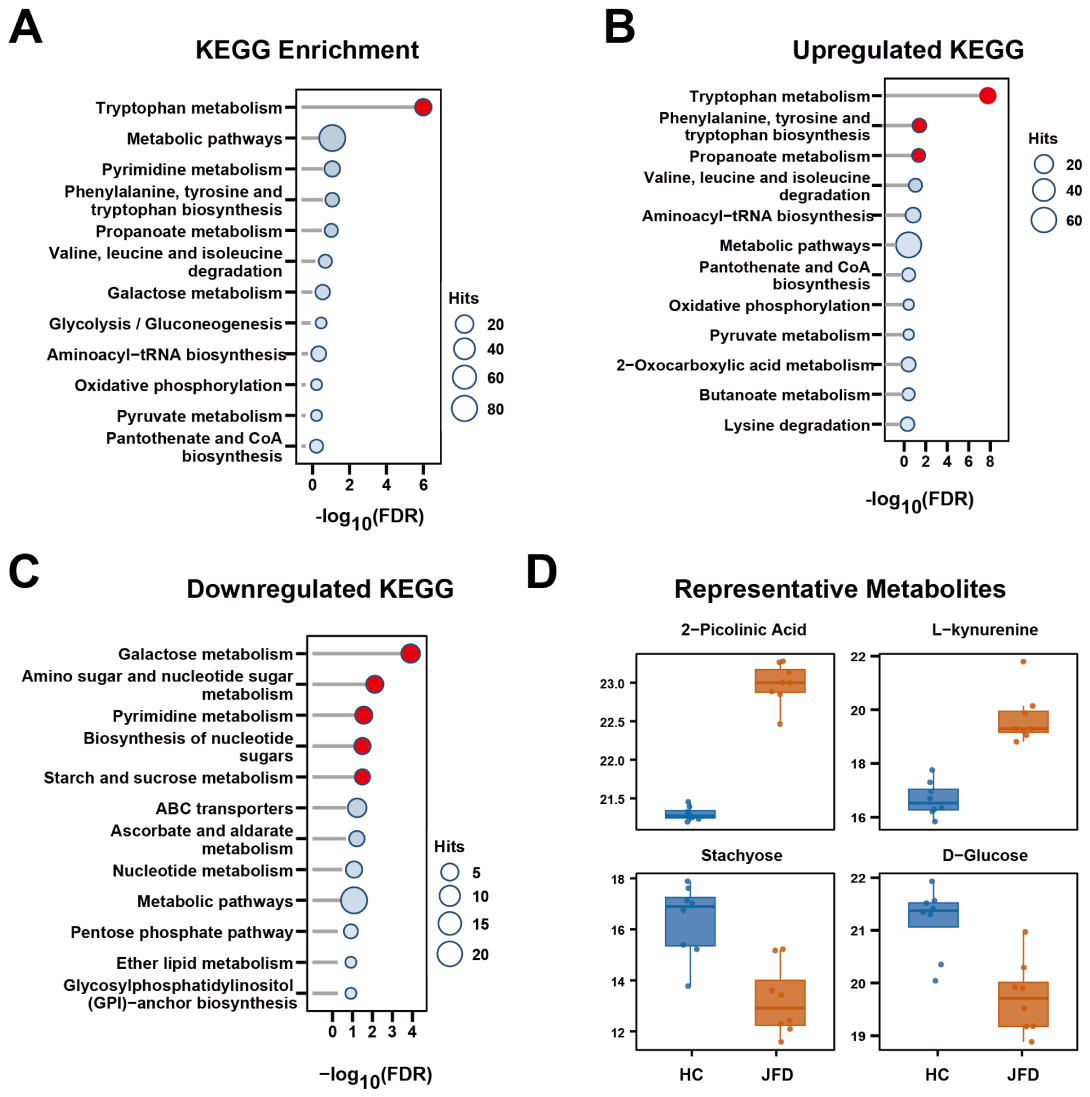
KEGG pathway enrichment analysis indicates elevated tryptophan-related metabolite changes and reduced sugar- and nucleotide sugar-related pathways in JFD bees. (**A**) KEGG enrichment analysis of all significant differentially abundant filtered metabolites in the HC versus JFD comparison. (**B**) KEGG enrichment analysis of filtered metabolites increased in JFD. (**C**) KEGG enrichment analysis of filtered metabolites decreased in JFD. (**D**) Relative abundances of representative retained metabolites showing directional changes consistent with the pathway-level results. In the enrichment plots, the *x*-axis indicates −log_10_(FDR), and dot size represents the number of matched metabolites (hits). Red dots indicate significantly enriched pathways (FDR < 0.05), whereas light-blue dots indicate pathways that did not meet this threshold. Gray horizontal lines connect each pathway to its corresponding dot and do not represent an additional statistic. In the boxplots, each point represents one sample. Boxes represent the interquartile range, horizontal lines within the boxes indicate the median, and whiskers extend to the most extreme values within 1.5 times the interquartile range.

To identify the major pathways represented by the differential metabolites, we performed KEGG enrichment analysis using the significant differentially abundant filtered metabolites. Across all significant metabolites, tryptophan metabolism showed the strongest pathway enrichment, with 14 matches among 16 annotated background metabolites and an FDR of $9.15\times 10^{-7}$ (Figure 2A). In honeybees, microbial regulation of tryptophan metabolism has been linked to gut–brain interactions and learning and memory behavior [27]. Other enriched pathways in the overall differential-metabolite set included glycolysis/gluconeogenesis, propanoate metabolism, phenylalanine, tyrosine and tryptophan biosynthesis, pyrimidine metabolism, and galactose metabolism, indicating that amino acid-related and carbon-flow-related changes co-occurred in JFD.

When significant metabolites were stratified by direction of change, metabolites increased in JFD were primarily enriched in tryptophan-related and related amino acid metabolic pathways. Tryptophan metabolism ranked first among the upregulated metabolites, with 14 matches among 16 annotated background metabolites and an FDR of 1.69 × 10^−8^. Phenylalanine, tyrosine and tryptophan biosynthesis was also significantly enriched (5/7; FDR = 0.0380), as was propanoate metabolism (4/5; FDR = 0.0451) (Figure 2B).

By contrast, metabolites decreased in JFD were significantly enriched in carbohydrate- and nucleotide sugar-related pathways. These included galactose metabolism (7/16; FDR = 1.23 × 10^−4^), amino sugar and nucleotide sugar metabolism (5/15; FDR = 0.00762), pyrimidine metabolism (5/21; FDR = 0.0273), biosynthesis of nucleotide sugars (4/15; FDR = 0.0318), and starch and sucrose metabolism (3/8; FDR = 0.0318) (Figure 2C). Additional top-ranked pathways displayed in the decreased subset included ABC transporters, ascorbate and aldarate metabolism, and nucleotide metabolism, but these did not reach FDR < 0.05. Representative boxplots further showed that 2-picolinic acid and L-kynurenine were higher in JFD, whereas stachyose and D-glucose were lower in JFD (Figure 2D).

### 3.3. RNA-Seq Reveals Limited Differential Expression but Reproducible Transcript-Level Remodeling in JFD Bees

**Figure 3:**
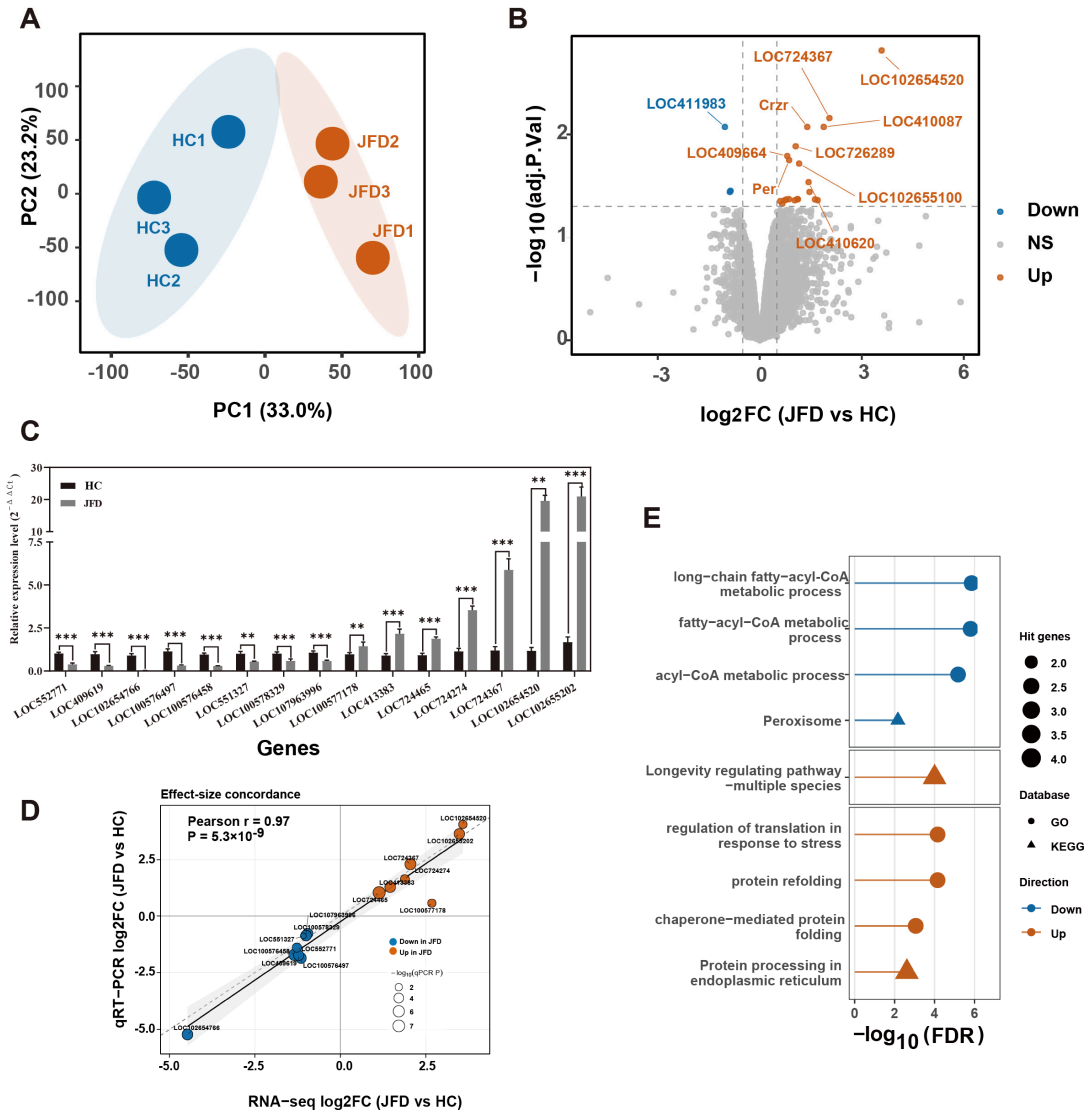
RNA-seq analysis identifies limited differential-expression changes but reproducible transcript-level remodeling in JFD bees. (**A**) Principal component analysis (PCA) based on the voom-transformed RNA-seq expression matrix showed a tendency toward separation between HC and JFD samples. Ellipses indicate the 95% confidence regions for each group. Blue points and ellipses represent HC samples, whereas orange points and ellipses represent JFD samples. (**B**) Volcano plot of differential gene expression between HC and JFD. Genes meeting both FDR < 0.05 and |log2FC| ≥ 0.5 are highlighted, and representative genes are labeled. The horizontal dashed line indicates FDR = 0.05, and the two vertical dashed lines indicate log_2_FC = −0.5 and 0.5. Solid colored leader lines connect gene labels to their corresponding points and do not represent an additional statistic. Orange indicates genes upregulated in JFD, blue indicates genes downregulated in JFD, and gray indicates genes that did not meet both thresholds. (**C**) qRT-PCR validation of 15 randomly selected genes. Black and gray bars represent HC and JFD samples, respectively. Bars show mean relative expression calculated using the 2^^−ΔΔCt^ method, and error bars indicate the standard deviation. Asterisks indicate statistical significance: ** *p* < 0.01 and *** *p* < 0.001. (**D**) Concordance between RNA-seq and qRT-PCR log_2_ fold-change estimates for the 15 validated genes. Each point represents one gene; orange and blue indicate genes upregulated and downregulated in JFD, respectively. Point size represents −log10(P) from the qRT-PCR comparison. The solid black line represents the fitted linear regression, the surrounding gray band indicates its 95% confidence interval, and the gray dashed line indicates the line of identity (y = x). The thin horizontal and vertical gray lines indicate zero fold change. (**E**) Functional enrichment analysis of upregulated and downregulated differentially expressed genes. The *x*-axis indicates −log10(FDR), and dot size represents the number of matched genes. Circles and triangles denote Gene Ontology (GO) and KEGG terms, respectively. Blue represents enrichment among genes downregulated in JFD, whereas orange represents enrichment among genes upregulated in JFD. Colored horizontal lines connect each term to its corresponding enrichment point and do not represent an additional statistic. HC, exposed asymptomatic jujube flower foragers; JFD, jujube flower disease.

To determine whether the metabolite-level changes were accompanied by transcriptional remodeling, we performed RNA-seq analysis in HC and JFD bees. Principal component analysis based on the limma-voom expression matrix showed a tendency toward separation between the two groups, and the volcano plot summarized the overall differential-expression structure. A total of 10,259 genes were detected, of which 23 met the thresholds of FDR<0.05 and $|{log}_{2}FC|\geq 0.5$. Among these 23 genes, 20 were increased and 3 were decreased in JFD (Figure 3A,B).

To assess the general concordance between RNA-seq and qRT-PCR measurements, we examined 15 randomly selected genes from the RNA-seq dataset by qRT-PCR. The direction of change was concordant between qRT-PCR and RNA-seq, and the effect-size estimates from the two assays were strongly correlated across the validated genes (r =0.97, $P\;=5.3\times 10^{-9}$), supporting the overall concordance between the two measurement methods for these selected genes (Figure 3C,D).

We next performed functional enrichment analysis separately for increased and decreased genes. Genes decreased in JFD were enriched mainly in long-chain fatty-acyl-CoA metabolic process, fatty-acyl-CoA metabolic process, acyl-CoA metabolic process, and peroxisome. By contrast, genes increased in JFD were enriched mainly in protein processing in the endoplasmic reticulum, protein refolding, chaperone-mediated protein folding, regulation of translation in response to stress, and the longevity-regulating pathways of multiple species (Figure 3E). These results suggest that although the number of differentially expressed genes was limited, the transcriptomic changes were concentrated in lipid/peroxisome-related processes and proteostasis/stress-related pathways rather than directly mirroring the dominant metabolite-level changes. 

### 3.4. Targeted Gene-Set Analysis Suggests Moderate Downregulation of the Sugar Supply/Storage Program in JFD Bees

**Figure 4:**
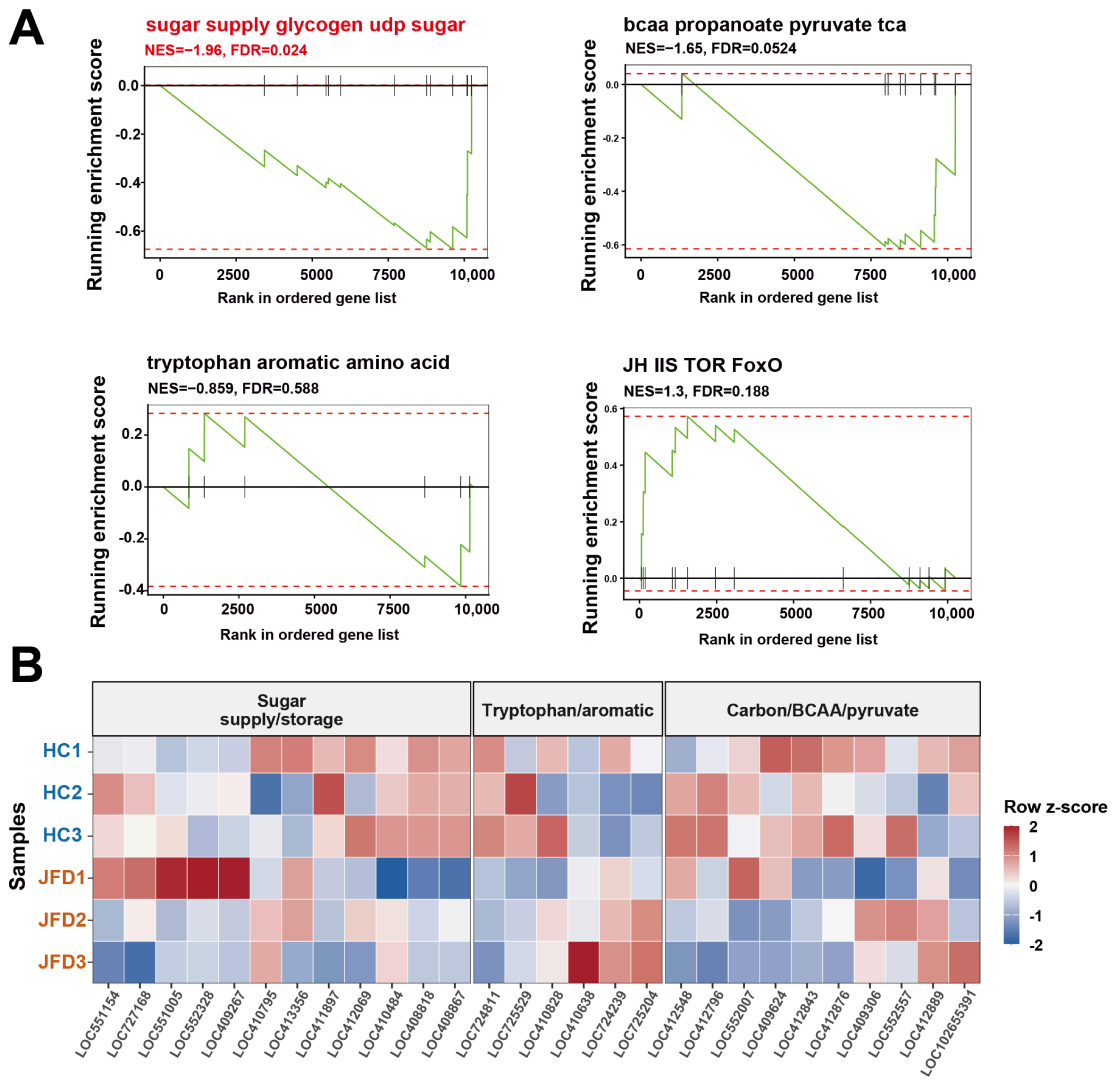
Targeted GSEA and heatmap visualization of curated transcriptomic gene sets. (**A**) Preranked gene-set enrichment analysis (GSEA) plots for four curated gene sets: sugar supply/storage, carbon/BCAA/pyruvate metabolism, tryptophan/aromatic amino acid metabolism, and JH/IIS/TOR/FoxO signaling. In each plot, the *x*-axis indicates the rank position of genes in the ordered gene list, and the *y*-axis indicates the running enrichment score. The green curve represents the running enrichment score, the solid black horizontal line marks an enrichment score of zero, and the red dashed horizontal lines indicate the maximum and minimum running enrichment scores reached along the ranked gene list. Black vertical tick marks indicate the positions of genes belonging to the corresponding gene set. NES and FDR values are shown as panel annotations. (**B**) Targeted heatmap of representative genes from the sugar supply/storage, tryptophan/aromatic amino acid, and carbon/BCAA/pyruvate gene sets. Columns represent genes, and rows represent RNA-seq samples. The color scale indicates z-score-standardized voom expression values for each gene across samples; red denotes relatively higher expression and blue denotes relatively lower expression. BCAA, branched-chain amino acid; JH, juvenile hormone; IIS, insulin/insulin-like signaling; TOR, target of rapamycin; FoxO, Forkhead box O.

Based on the directional metabolomic results showing increased tryptophan/aromatic amino acid-related metabolism and decreased sugar- and nucleotide sugar-related pathways, we next constructed corresponding gene sets to evaluate transcript-level gene-set changes associated with sugar supply/storage, tryptophan/aromatic amino acids, carbon flow, and JH/IIS/TOR/FoxO-related nutrient signaling. The genes included in these curated gene sets are summarized in Table 2. GSEA showed that the sugar supply/storage gene set had moderate negative enrichment in JFD bees (NES = −1.96; FDR = 0.024), and was the only predefined transcriptomic gene set reaching FDR < 0.05. The carbon-flow-related gene set showed a borderline negative trend (NES = −1.65; FDR = 0.0524), whereas the JH/IIS/TOR/FoxO-related gene set did not reach the FDR threshold (NES = 1.30; FDR = 0.188). The tryptophan/aromatic amino acid gene set was not enriched at the transcriptomic level (NES = −0.859; FDR = 0.588) (Figure 4A).

To further examine the gene-level expression changes within these gene sets, we generated a targeted heatmap of representative genes from the sugar supply/storage, tryptophan/aromatic amino acid, and carbon/BCAA/pyruvate-related sets. Most genes in the sugar supply/storage-related group tended to show lower expression in JFD samples overall. Among the 12 genes in this set, HK, LOC408867, LOC410484, and LOC411897 showed particularly consistent decreases, with ${log}_{2}FC$ values of −0.8188, −0.4371, −0.4345, and −0.2716, respectively. By comparison, the tryptophan/aromatic amino acid-related set showed more localized increases, whereas the BCAA/propanoate/pyruvate-related set showed a more modest and heterogeneous downward tendency (Figure 4B).

### 3.5. Exploratory Enzyme–Metabolite Covariation Within the Sugar Supply/Storage Axis

To further examine gene–metabolite relationships within the sugar supply/storage-related gene set, we focused on direct biochemical relationships between encoded enzymes and measured sugar-related metabolites. Five representative enzyme–metabolite pairs were selected for visualization: Pgm1–D-glucose-6-phosphate, trehalase–D-glucose, Pgi–D-glucose-6-phosphate, Tps1–UDP-glucose, and HK–D-glucose. These pairs were selected because the corresponding enzymes are directly linked to the displayed metabolites as substrates or products within the sugar supply/storage-related pathway context.

**Figure 5:**
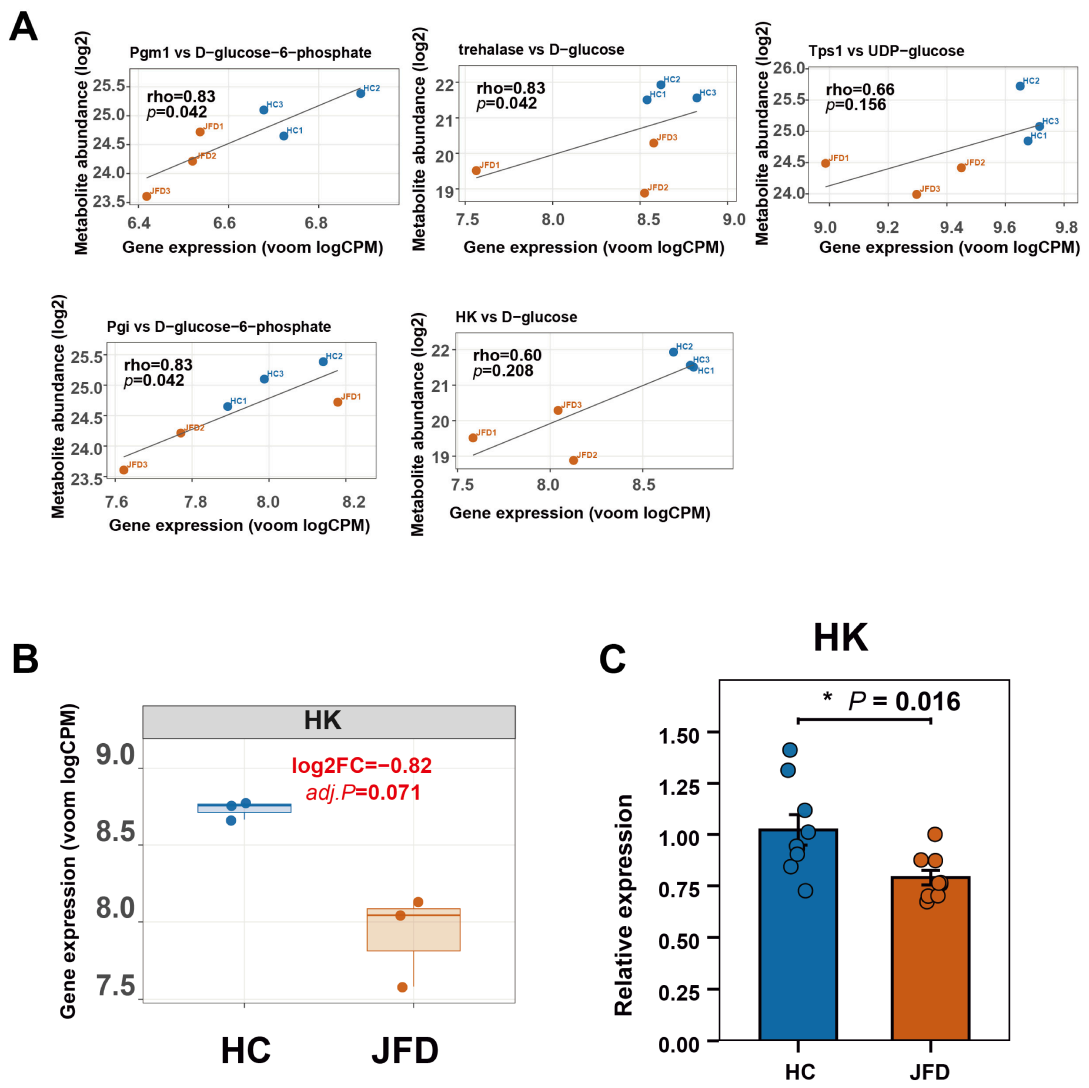
Direct enzyme–metabolite covariation and HK follow-up validation within the sugar supply/storage axis. (**A**) Scatter plots of five representative gene–metabolite pairs selected based on direct enzyme–metabolite correspondence: Pgm1–D-glucose-6-phosphate, trehalase–D-glucose, Pgi–D-glucose-6-phosphate, Tps1–UDP-glucose, and HK–D-glucose. Each point represents one matched sample, and sample labels are shown. Spearman’s ρ and nominal *p* values are presented as descriptive annotations. (**B**) RNA-seq expression distribution of HK in HC and JFD samples. Expression values are shown as voom-derived logCPM values overlaid with individual sample points; the RNA-seq log_2_FC and adjusted *p* value are indicated. (**C**) Targeted qRT-PCR validation of HK expression in HC and JFD bees. Eight biological replicates were analyzed per group (*n* = 8), with three technical replicate reactions performed for each biological sample. Technical replicates were averaged before statistical analysis, and the biological replicate means were compared using a two-sided unpaired Student’s *t*-test.

Across the six matched samples, all five pairs showed positive sample-level associations, including Pgm1–D-glucose-6-phosphate (Spearman’s ρ = 0.83, *p* = 0.042), trehalase–D-glucose (Spearman’s ρ = 0.83, *p* = 0.042), Pgi–D-glucose-6-phosphate (Spearman’s ρ = 0.83, *p* = 0.042), Tps1–UDP-glucose (Spearman’s ρ = 0.66, *p* = 0.156), and HK–D-glucose (Spearman’s ρ = 0.60, *p* = 0.208) (Figure 5A). Individual sample labels were shown in the scatter plots to allow for visual inspection of sample-level influence.

To further evaluate whether these associations were driven by individual samples, we performed leave-one-out sensitivity analysis for the same five gene–metabolite pairs. For each pair, Spearman’s ρ was recalculated after removing one sample at a time. The correlations remained positive after omission of individual samples, and no direction reversal was observed (Supplementary Figure S3A). These findings suggest that the directions of the displayed associations were not determined solely by a single sample, although the analyses remain exploratory because of the small matched sample size.

We then examined the expression distributions of the five genes involved in these direct enzyme–metabolite pairs. All five genes showed lower expression trends in JFD samples, with log_2_FC values of −0.27 for Pgm1, −0.14 for Pgi, −0.82 for HK, −0.44 for Tps1, and −0.43 for trehalase (Supplementary Figure S3B). Among them, HK showed the largest RNA-seq decrease and approached, but did not reach, the FDR < 0.05 threshold after multiple-testing correction (FDR = 0.071) (Figure 5B). Targeted qRT-PCR analysis further supported the reduced expression trend of HK in JFD bees, consistent with the RNA-seq result (Figure 5C).

## 4. Discussion

This study integrated widely targeted metabolomics and RNA-seq to characterize molecular alterations associated with JFD in honeybees. Our findings reveal two related molecular patterns: tryptophan-related metabolite changes were most prominent at the metabolite level, whereas sugar supply/storage-related changes showed partial multi-omics support. Although tryptophan metabolism represented the strongest enrichment in the metabolomic analysis, the direction-stratified metabolite results and targeted transcriptomic analysis together pointed to reduced carbohydrate- and nucleotide sugar-related changes as a partially supported cross-layer finding. Within this sugar-related context, HK was retained as a putative follow-up gene.

Previous work on JFD has mainly emphasized gut-localized responses, especially midgut transcriptional changes related to antioxidant and detoxification processes [5]. Broader studies of the bee gut microbiota likewise show that gut bacteria can shape honeybee health, metabolism, and behavior [28,29] and can modify the host metabolic environment through microbial saccharide breakdown and fermentation [30]. Our results do not contradict the relevance of these local responses. Rather, they extend this picture by suggesting that disturbance of energy-related metabolism is also a prominent component of the present dataset. This interpretation is further compatible with evidence that host–microbiome interactions can contribute to the metabolism of plant-derived toxins in bees [31]. Taken together, these observations support a view in which JFD may involve both local tissue responses and broader metabolite abundance alterations.

Our analyses further suggest that the sugar supply/storage axis deserves particular attention because it is consistent with core features of honeybee nutritional physiology. Honeybees depend heavily on nectar-derived carbohydrates to sustain flight and foraging activity, and direct physiological studies show that foraging metabolism is tightly coupled to energetic demand and reward conditions [32,33]. At the behavioral level, foraging decisions are strongly influenced by nectar reward [34], whereas at the physiological level, hemolymph sugar levels and trehalose metabolism are closely linked to individual energy status [8,9,35]. In addition, dominant gut bacteria such as Gilliamella can metabolize a broad range of sugar substrates [36], supporting coordinated roles of the host and microbiota in carbohydrate use. In the present dataset, JFD was associated with an overall reduction in sugar- and nucleotide sugar-related pathways, accompanied by decreases in representative metabolites such as D-glucose and stachyose. At the transcriptomic level, the sugar supply/storage gene set showed the strongest negative enrichment among the predefined gene sets. Taken together, these observations support the interpretation that sugar-related metabolic alteration is partially supported across omics layers in the present dataset, rather than representing a single pathway that is equally prominent across both omics layers.

A cautious working interpretation is therefore that the sugar-related alteration may reflect reduced capacity to sustain high-demand energy use rather than a nonspecific shift in metabolite abundance alone. In honeybees, flight depends primarily on hexose oxidation, and key flight-muscle enzymes including hexokinase can operate close to their maximal flux during sustained activity; age- and caste-related increases in flight performance are also accompanied by increased maximal activities of hexokinase, phosphofructokinase, and pyruvate kinase [10,11]. More broadly across insects, reduced glycolytic function in neural tissue can impair olfactory memory, and defects in ATP production or ATP-dependent ion homeostasis can compromise neurotransmission under repeated stimulation [37,38].

However, the present cross-sectional design cannot determine whether the sugar-related alteration is an upstream contributor to JFD development or a secondary physiological response after symptom onset. The observed decreases could reflect impaired carbohydrate acquisition or utilization that precedes the symptoms, but they could also result from altered nectar intake, foraging behavior, crop or gut contents, digestion, microbial processing, or disease-associated changes in energy use. Therefore, these findings indicate a JFD-associated sugar-related metabolic state rather than establishing impaired carbohydrate metabolism as the primary cause of JFD.

Tryptophan-related metabolites represented the most prominent metabolite-level change in JFD. Tryptophan metabolism comprises several physiologically distinct branches in insects, including the kynurenine, serotonin, and microbial indole pathways. Tryptophan-derived signaling can intersect with insect immunity: serotonin modulates hemocyte phagocytosis, while the aryl hydrocarbon receptor, which can respond to kynurenine and microbial indole metabolites, participates in immune homeostasis in mosquitoes [39,40]. Kynurenine-pathway intermediates also exert metabolite-specific effects on oxidative homeostasis. In Drosophila, increased 3-hydroxykynurenine and xanthurenic acid enhanced light-induced retinal damage, whereas kynurenine and kynurenic acid showed protective effects under the same stress context [41]. Tryptophan is also a precursor for serotonin synthesis through tryptophan hydroxylase and DOPA decarboxylase, linking tryptophan availability and metabolism to serotonergic signaling [42]. In honeybees, gut Lactobacillus-mediated conversion of tryptophan into indole derivatives has been linked to improved learning and memory through aryl hydrocarbon receptor activation, and pharmacological blockade of serotonergic signaling impaired trace conditioning [27,43]. These findings indicate that tryptophan metabolism may influence immune, redox, neural, and behavioral physiology in insects, although the direction and biological consequence depend on the specific metabolic branch and metabolite involved. In the present dataset, metabolites such as kynurenine were significantly increased, but the corresponding tryptophan/aromatic amino acid gene set did not show overall transcriptomic enrichment. This dissociation suggests that the observed pathway-level alteration may be shaped more strongly by metabolic regulation, microbial input, substrate availability, post-transcriptional processes, or differences in gut or crop contents than by coordinated transcriptional induction. Accordingly, the tryptophan-related changes observed here are best interpreted as a dominant metabolome-level JFD-associated alteration. The present data do not establish which immune, oxidative, neural, or behavioral processes, if any, are affected by this alteration, and these possibilities require direct functional testing.

A similar point applies to the BCAA/propanoate/pyruvate-related changes. In this pathway, the consistency between metabolite and transcript levels was weaker, indicating that the relationship between these two molecular layers is not one-to-one. Metabolite levels are jointly shaped by multiple factors, including metabolic flux, substrate supply, transport, and enzyme activity, and therefore need not mirror transcript abundance directly [44,45]. The observed changes may therefore reflect altered carbon-flow states or accompanying metabolic variation, and their relationship to the major tryptophan-related and sugar-related alterations requires further validation.

This study also has several limitations. First, part of the metabolite identification was based on database annotation rather than validation with authentic standards [46,47]. Accordingly, these compounds should be interpreted as putatively annotated metabolites, and uncertainty may remain because structurally similar compounds or isomers can produce overlapping spectral features. Although annotation-based filtering removed features with clear exogenous annotations, irrelevant chemical categories, or putative plant-derived origins, it cannot eliminate annotation errors or definitively determine the biological origin of every detected compound. Therefore, pathway-level patterns should be interpreted more confidently than the identity or biological role of any individual unconfirmed metabolite, and key compounds require targeted validation using authentic standards. Because whole bee extracts were used for metabolomic profiling, some metabolite differences may still reflect floral-source compounds, dietary exposure, crop/gut contents, microbial metabolism, absorption, clearance, or compound transformation rather than direct host biosynthesis alone. In addition, the HC group consisted of active foragers collected from jujube flowers and should therefore be interpreted as exposed asymptomatic foragers rather than unexposed healthy controls. This sampling strategy reduced variation in floral exposure and foraging context, but it cannot distinguish disease mechanisms from individual tolerance, behavioral differences, different levels of jujube nectar intake, or pre-symptomatic baseline variation. Because JFD classification was based on field symptoms and no pathogen-specific or toxicological screening was performed, alternative diseases or intoxications producing overlapping clinical signs could not be completely excluded. Accordingly, JFD should be interpreted in the present study as a field-defined bloom-associated syndrome rather than an etiologically confirmed condition. Furthermore, only one individual was sampled from each colony, and the HC and JFD individuals originated from different colonies. Although this design avoided repeated sampling of multiple individuals from the same colony, it did not permit estimation of within-colony variability or separate statistical adjustment for colony-level effects. Consequently, unmeasured colony-level genetic, environmental, or management differences may have contributed to the observed group differences. The sampled colonies were not genotyped, and their genetic backgrounds were therefore not fully controlled. Future studies should include controlled jujube-nectar feeding, direct measurement of nectar intake and crop load, and time-series sampling before and after symptom onset. Additionally, the RNA-seq sample size was limited, and the enzyme–metabolite correlation analysis was based on only six matched samples. These relationships should therefore be interpreted strictly as exploratory indicators of sample-level covariation rather than confirmatory evidence of gene–metabolite regulation. Although the leave-one-out analysis was used to assess sensitivity to individual samples, it does not overcome the limited statistical power of the small sample size. Larger independent datasets are required to validate these relationships. Moreover, transcriptional changes are not equivalent to metabolic flux or enzyme activity, and the present data do not establish a causal or sequential relationship among the tryptophan-related alterations, sugar-related alterations, and JFD progression. Any proposed connection among these features should therefore be regarded as a working hypothesis rather than an established biological mechanism. Future work should therefore focus on validating sugar supply/storage-related candidate genes with larger sample sizes, targeted metabolomics, enzyme activity assays, tissue-specific or controlled-feeding metabolomics, microbiota intervention, and exposure experiments, so that the stage-specific roles of these changes during disease development can be more clearly distinguished.

## 5. Conclusions

JFD in honeybees is associated with molecular alterations characterized by increased tryptophan-related metabolite abundance and reduced carbohydrate- and nucleotide sugar-related metabolite abundance. Targeted transcriptomic analysis provided moderate support for negative enrichment of the sugar supply/storage gene set, whereas the tryptophan-related metabolite changes were not mirrored by transcriptomic gene-set enrichment. HK was retained only as a candidate for future functional validation within the sugar-related axis. Overall, this study provides a multi-omics framework for characterizing JFD-associated metabolic alterations and identifies candidate metabolic pathways and genes for future mechanistic investigation, rather than definitive evidence of disease causation.

## Figures and Tables

**Table 1:** Primer sequences used for the qRT-PCR validation.

Gene ID	Forward Primer (5′–3′)	Reverse Primer (5′–3′)	Amplification Efficiency	R^2^
LOC406122	ACTACGGCCGAACGTGAAAT	GGAAAAGAGCCTCGGGACAA	100.04%	0.9984
LOC408804	GCCACGCTACGAGACGAATG	ACCGAATGTCGAGGGAAGGA	105.03%	0.9901
LOC409619	GCGAGAGCAGCGGTTTATTG	GCGCTTCGATACTTCTCCCA	90.91%	0.9987
LOC413383	GCGTTGTCCCTTCTTCTCCA	CATCGCCTGGCCTCCATATT	101.87%	0.9940
LOC551327	TCGGGATTGTCTTCGTTGGG	TATCCGGCACATGGATTCGG	97.08%	0.9941
LOC552771	ATTCCAGCAACGATTGAGGC	CCTTGCCTTATACCTCCGTCC	102.43%	0.9900
LOC724274	GCACGGTTGGATTTCACGTC	AGCAGTGATAGTGAGAACGCC	94.21%	0.9993
LOC724367	GTCGACCATTACAGCGGACA	TGTTTCGCCTCTACCACGAC	98.11%	0.9932
LOC724465	GATGCTGGAGAGGAAGAGGC	TGAGCTCGAGAATGTCAGCC	104.53%	0.9909
LOC100576458	CACAATGGGCTTGATACGGC	AGTCAATGTGCACCCCTGTC	99.00%	0.9975
LOC100576497	TTCAAGGCCGTTCAATTGCG	ACATAAAGACCCCATCGCGT	92.16%	0.9961
LOC100577178	TTGCGAACGCGATTAACAGG	TCTGCTGATCCCCGATACCT	104.07%	0.9918
LOC100578329	TGGCTGCAACTGTCCGATTC	TCCGTACAAGGTGCAAATGC	97.39%	0.9979
LOC102654766	TGGTCAAGCGGCAACTGTTA	TTGGCTGTATGGCATGTGGT	92.90%	0.9943
LOC102654520	GGACAAATCCAGGTAACAGCG	GCCACGAAAAATCGTTTGCC	91.00%	0.9964
LOC102655202	GTTTCGCGTGGTTGTCTGTT	TGGTGTGCTCGATTTGGTGA	93.43%	0.9992
LOC107963996	GCTACGAATTCACGCACACC	GCACGGATTGTTAGACGCAC	103.45%	0.9953

Primer sequences used for the qRT-PCR validation, including gene ID, forward primer (5–3), reverse primer (5–3), amplification efficiency, and R2.

**Table 2:** Curated gene sets used for the targeted transcriptomic analyses shown in Figure 4.

Gene Set	Genes
sugar_supply_glycogen_udp_sugar	LOC408818, LOC408867, LOC409267, LOC410484, LOC410795, LOC411897, LOC412069, LOC413356, LOC551005, LOC551154, LOC552328, LOC727168
bcaa_propanoate_pyruvate_tca	LOC102655391, LOC409306, LOC409624, LOC412548, LOC412796, LOC412843, LOC412876, LOC412889, LOC552007, LOC552557
JH_IIS_TOR_FoxO	LOC406152, LOC408438, LOC409393, LOC409725, LOC410732, LOC411297, LOC413430, LOC551179, LOC551500, LOC551668, LOC724216, LOC725039, LOC727091
tryptophan_aromatic_amino_acid	LOC410638, LOC410828, LOC724239, LOC724811, LOC725204, LOC725529

Curated gene sets used for the targeted transcriptomic analyses shown in Figure 4. LOC identifiers correspond to annotated Apis mellifera gene loci. BCAA, branched-chain amino acid; JH, juvenile hormone; IIS, insulin/insulin-like signaling; TOR, target of rapamycin; FoxO, Forkhead box O.

## Data Availability

The raw RNA-seq data generated in this study are publicly available in the Genome Sequence Archive (GSA) under accession number CRA042444 (https://ngdc.cncb.ac.cn/gsa/search?searchTerm=CRA042444) (accessed on 21 July 2026). The processed metabolite abundance data and associated metadata are publicly available in Zenodo at https://doi.org/10.5281/zenodo.21457121. Additional data supporting the findings of this study are provided within the article and its Supplementary Materials.

## Supplementary Materials

The following supporting information can be downloaded at: https://www.mdpi.com/article/10.3390/biology15151236/s1, Figure S1: Principal component analysis of biological samples and pooled quality-control injections; Figure S2: Metabolite annotation-based filtering workflow; Figure S3: Sensitivity analyses and RNA-seq expression distributions for the exploratory enzyme–metabolite analysis; Table S1: Information and coefficients of variation for the eight internal standards; Table S2: Sample-level RNA-seq sequencing and alignment quality metrics; Table S3: Filtered annotated metabolites retained for downstream analysis and metabolite features excluded during annotation-based filtering; Table S4: Complete list of significantly altered metabolites between JFD and HC.

## Abbreviations

The following abbreviations are used in this manuscript:

BCAA	branched-chain amino acid
FDR	false discovery rate
FoxO	Forkhead box O
GO	Gene Ontology
GSEA	Gene-set enrichment analysis
HC	exposed asymptomatic jujube flower foragers
HK	hexokinase
HMDB	Human Metabolome Database
IIS	insulin/insulin-like signaling
JFD	jujube flower disease
JH	juvenile hormone
KEGG	Kyoto Encyclopedia of Genes and Genomes
LC-QTOF-MS/MS	liquid chromatography–quadrupole time-of-flight–tandem mass spectrometry
MRM	multiple reaction monitoring
NES	normalized enrichment score
PCA	principal component analysis
QC	quality control
qRT-PCR	quantitative real-time polymerase chain reaction
RNA-seq	RNA sequencing
TOR	target of rapamycin
UPLC-MS/MS	ultra-performance liquid chromatography–tandem mass spectrometry
