# Supplementary material for: Integrated Metabolomic and Transcriptomic Analyses Identify Elevated Tryptophan Metabolism and Altered Sugar Homeostasis Associated with Honeybee Jujube Flower Disease
Source: Biology (Basel). 2026 Jul 26;15(15):1236. doi: 10.3390/biology15151236 (PMC13465942; doi:10.3390/biology15151236)
Supplement: Supplementary file 1 [file biology-15-01236-s001.zip › Supplementary Figures S1-S3.pdf]

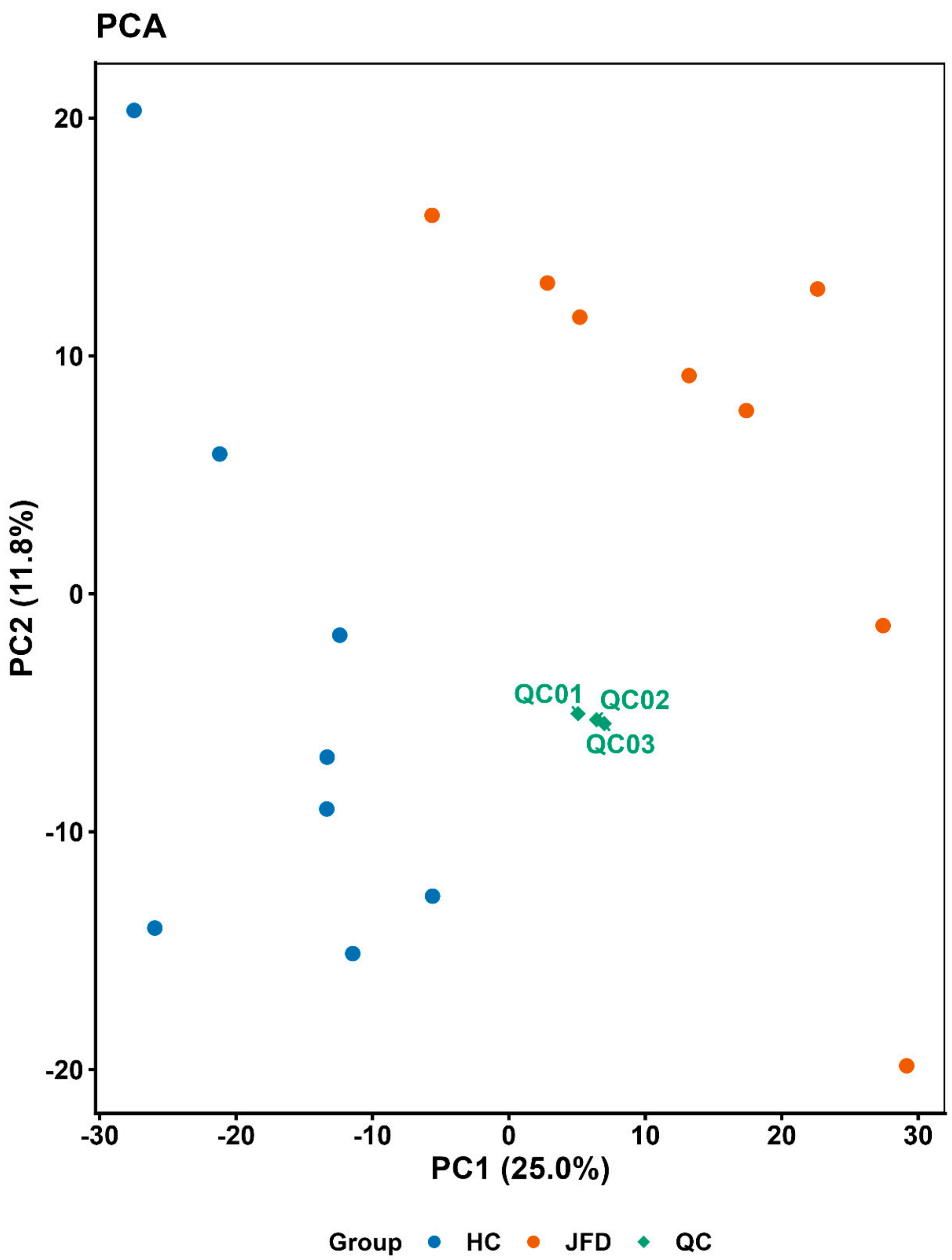

Supplementary Figure S1. Principal component analysis of biological samples and pooled quality-control injections. PCA was performed using the log2-transformed abundance matrix of the retained metabolites across eight HC samples, eight JFD samples, and three pooled QC injections. Each point represents one biological sample or one pooled QC injection. The three QC injections clustered closely in PCA space. HC, asymptomatic honeybees collected during jujube flowering; JFD, honeybees showing jujube flower disease symptoms; QC, pooled quality-control injection.

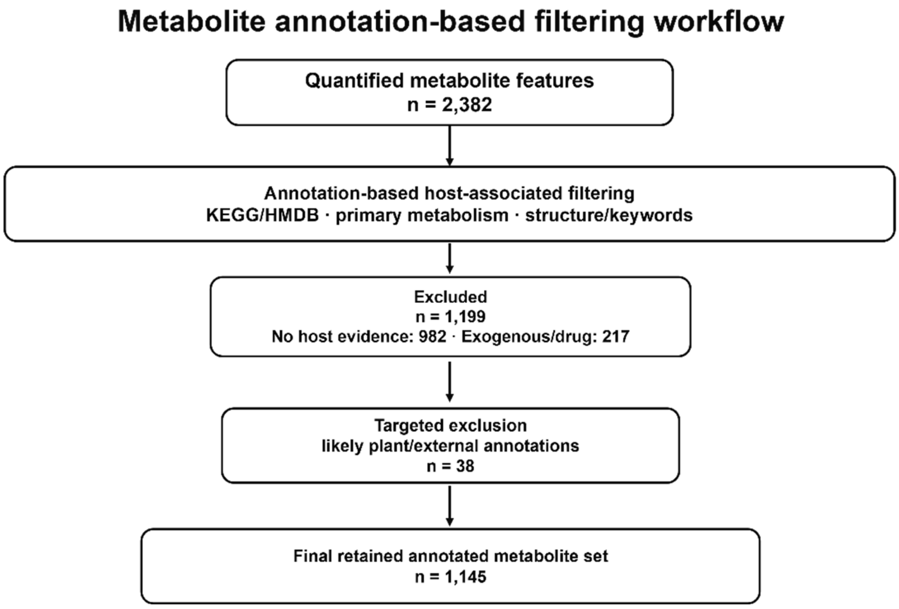

Supplementary Figure S2. Metabolite annotation-based filtering workflow. A total of 2,382 quantified metabolite features were subjected to annotation-based filtering using KEGG and HMDB information, primary metabolic classification, and structure- and keyword-based criteria. In the first filtering step, 1,199 features were excluded, including 982 metabolites lacking sufficient evidence of host association and 217 metabolites annotated as exogenous compounds or drugs. A further 38 metabolites with likely plant-derived or other external annotations were removed through targeted review. The final retained dataset comprised 1,145 putatively annotated, host-associated metabolites used for downstream analyses.

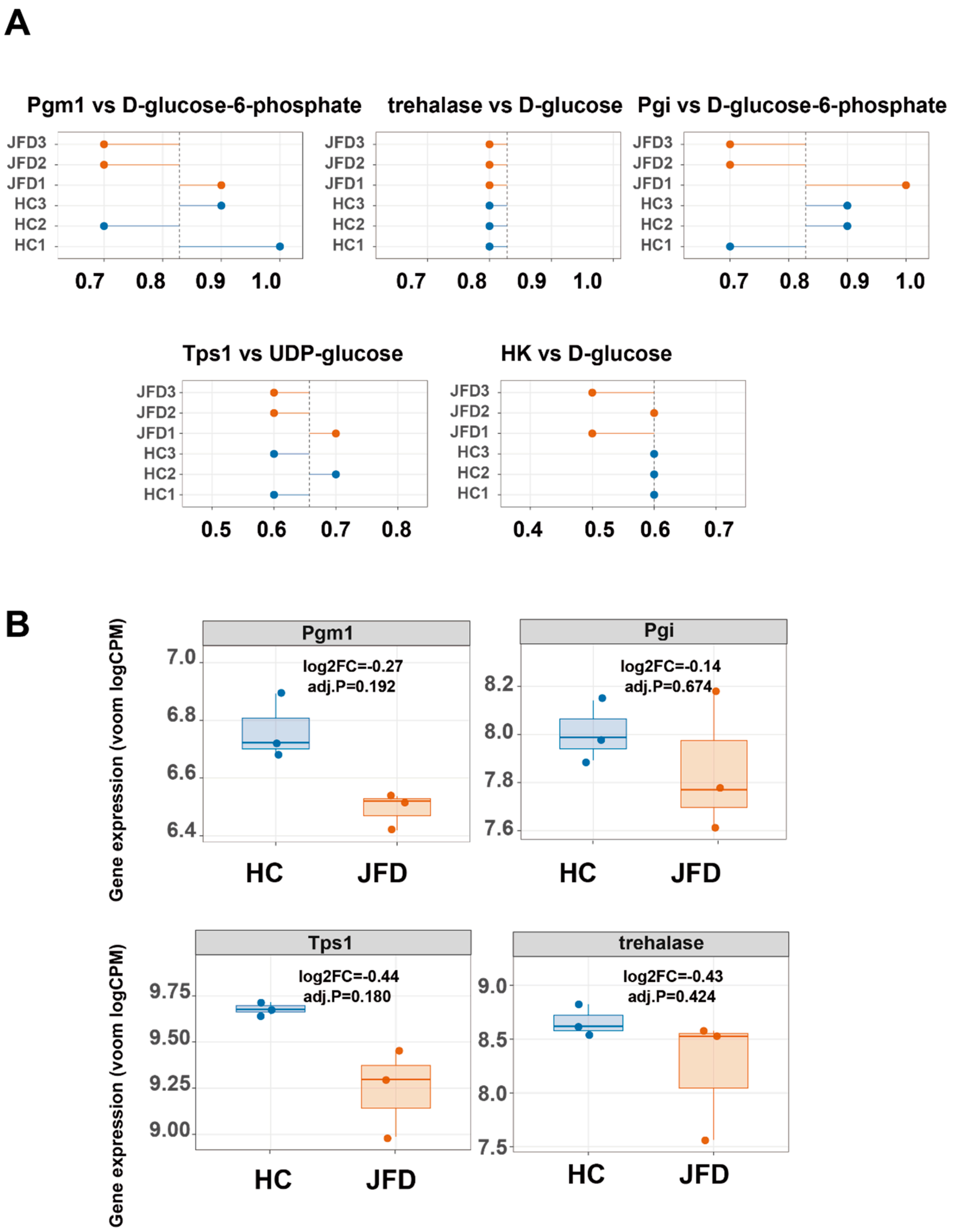

Supplementary Figure S3. Sensitivity analyses and RNA-seq expression distributions for the exploratory enzyme–metabolite analysis. (A) Leave-one-out sensitivity analysis for the five displayed enzyme–metabolite pairs. Each point represents Spearman's ρ recalculated after omission of one matched sample, and the dashed vertical line indicates the full-sample Spearman's ρ. (B) RNA-seq expression distributions of Pgm1, Pgi, Tps1, and trehalase in HC and JFD samples. Expression values are shown as voom-derived logCPM boxplots overlaid with individual sample points; RNA-seq log2FC and adjusted P values are indicated.
